# Exploring the socio-ecological levels for prevention of sexual risk behaviours of the youth in uMgungundlovu District Municipality, KwaZulu-Natal

**DOI:** 10.4102/phcfm.v10i1.1590

**Published:** 2018-03-07

**Authors:** Nelisiwe Khuzwayo, Myra Taylor

**Affiliations:** 1 School of Nursing and Public Health Medicine, University of KwaZulu-Natal, South Africa

## Abstract

**Background:**

Prevention of youth sexual risk behaviour among the youth in uMgungundlovu District Municipality continues to be a primary challenge for public health and health promotion. Current prevention interventions are targeted at an individual level, whilst youth behaviour is influenced by many social and environmental factors.

**Aim:**

The aim of the study was to explore the factors influencing sexual risk behaviours of the youth at different socio-ecological levels in uMgungundlovu District Municipality.

**Methods:**

An explorative and descriptive qualitative study design was used, using in-depth interviews and focus group discussions for data collection. A framework analysis was used to develop themes derived from the socio-ecological theory.

**Results:**

Four themes were identified that influence youth to engage in sexual risk behaviours: (1) individual factors, related to role modelling behaviour, gender and negative stereotypes towards females; (2) the microsystem in which youth function including the influence of family and peers; (3) the exo-system comprising the disadvantaged socio-economic status of the communities where the youth live; and (4) the macrosystem where negative social norms were reported to influence youth health outcomes.

**Conclusion:**

Sexual risk behaviour among youth in uMgungundlovu is influenced by many factors at multiple social levels. Interventions directed at these multiple levels are needed urgently.

## Introduction

With high-risk sexual behaviours evident among many South African youth aged 14–24 years, numerous interventions have been implemented intending to promote their sexual health.^1,2^ The ability of youth to protect themselves from unplanned pregnancy,^3^ intergenerational sex, intimate partner violence,^4^ human immunodeficiency virus (HIV) and acquired immune deficiency syndrome (AIDS) and sexually transmitted infections (STI) remains a challenge.^5^ The risk factors that have been found to increase this vulnerability to HIV, other STIs and unplanned teenage pregnancy include poor decision-making and peer pressure,^6^ social norms and gender-based violence,^7^ early sexual debut without protection,^5^ concurrent partners,^8^ intimate partner violence^9^ and intergenerational sex.^10^ Owing to the complexity of changing the above-mentioned factors influencing these risky behaviours among the youth, the South African government, civil organisations and non-governmental organisations have developed numerous educational programmes.^1,11^ However, most of these programmes^12^ mainly revolve around provision of information about sexual health.^13,14^ These programmes have used a variety of communication approaches such as different media, including television, radio, billboards and newspapers, as well as face-to-face information sharing and teaching strategies such as HIV peer education programmes and the Life Orientation learning area, which was introduced in South African public schools in 1995. Evaluation of the impact of the current interventions on risk reduction among the youth in southern Africa has shown limited success.^15^ Most of the face-to-face education programmes for the youth are implemented in school settings, where youth spend most of their time, and evidence shows that many HIV peer education programmes and Life Orientation learning area are operating in a vacuum,^16^ not linked to collaborative community structures and government services.^17,18^ The challenge arises when the situation requires youth to be proactive to get health and social services; they struggle to navigate within the clinics, schools and department of social development and non-governmental organisations to obtain the required services. The high prevalence of HIV and STIs among youth, and high rates of teenage pregnancy indicate a problem in accessing relevant prevention services and the poor social environmental support.^5^ Current approaches to risk reduction behaviour have not resulted in adequate positive health outcomes, and as a result, many youth remain affected by HIV and AIDS, unplanned teenage pregnancy, substance abuse and gender-based violence.^19^ Presently, young people between the ages of 14 and 24 years carry the highest burden of HIV and AIDS in South Africa.^20^

Interventions to reduce vulnerability of young people to risk sex behaviours have been seen as a cornerstone worldwide,^21^ with most taking place in specific settings such as schools,^22^ communities,^23,24^ and in mass media.^25,26^A systematic review assessing the effectiveness of HIV prevention interventions in changing sexual behaviour of young people (10–25 years) in sub-Saharan Africa found that there were no positive effects on sexual behaviour and that condom use at last sexual encounter only increased among males (relative risk = 1.46; 95% confidence interval = 1.31–1.64).

A reduction was reported in herpes simplex virus-2 but not in HIV incidence.^15^ Another systematic review that evaluated the effectiveness of mass media campaigns^27^ aiming at providing education on HIV and sexual health in developing country showed mixed results.^25^ This was a review of 15 mass media interventions addressing HIV-related behaviours among young people, with 11 based in Africa, 2 in Latin America and 1 examining a programme that took place in 44 developed and developing countries. These studies assessed change in knowledge, self-efficacy to pursue preventative behaviours, and perception of personal risk and social norms. Community trials of comprehensive approaches involving schools in Uganda,^27^ Tanzania^28^ and Zimbabwe^26^ failed to significantly reduce HIV incidence in young people. This is regardless of the early evidence from the high income and low and middle income countries, which suggested an important role of school-based interventions in increasing young people’s knowledge of sexuality, reproductive health and HIV.^29^ In South Africa, only two studies showed promising results in reducing reported related risk behaviours and these studies addressed structural factors and social influences underlying HIV risk.^23,24^ These results support the new socio-ecological approaches in public health that promote comprehensive intervention in addressing health matters. Young people are surrounded by layers of relationships which should be targeted as well; the inner circle is the microsystem in which young people have direct, face-to-face relationships with significant people such as parents, siblings, extended family members, sexual partners and friends; an outer circle comprises people who are indirectly involved in the development of young people such as family health care workers and parent’s employer (exo-systemic), and the prevailing cultural and economic conditions of the society (macrosystem).^30^

Owing to continuing poor health outcomes in youth, the aim of this study was to explore socio-ecological factors influencing youth risk behaviours in uMgungundlovu District Municipality.

## Methods

### Study design

This study forms part of the bigger study (results are reported elsewhere). The article reports phase one which was a descriptive qualitative study. A qualitative approach was most suitable to explore the factors influencing youth’s risk behaviour in uMgungundlovu District Municipality, KwaZulu-Natal Province.

### Study setting

UMgungundlovu District Municipality has a population of 1 017 763 and is the second largest municipality in KwaZulu-Natal. The official unemployment rate in KwaZulu-Natal Province was 27.1% in 2016. The municipality comprises seven local regions: uMngeni, uMshwati, uMkhambathini, uMsunduzi, Richmond, Impendle and Mpofana. Most of the people are Africans; 79% of the people speak isiZulu. There are slightly more females (52%) than males, and young people between the ages of 10 and 24 years constitute about 30% of the population.

The study selected facilitators and youth volunteers that were working with Operation Sukuma Sakhe (OSS). OSS is the first collaborative intervention in KwaZulu-Natal and, thus far, the only government service delivery model in which government departments, communities, social networks and non-governmental organisations sit together in ‘war-rooms’ (community halls) to discuss the social determinants of health and to develop coordinated plans for delivery of services. These facilitators were expected to attend war-rooms to share and give reports on behavioural change interventions for young people.

### Study population and sampling

The participants were youth facilitators and volunteers working within the district municipality. All of the participants speak Zulu. Participants were recruited through the Office of the Premier of KwaZulu-Natal and The AIDS Foundation. All the facilitators that were previously employed by the Office of the Premier in KwaZulu-Natal and currently employed by The AIDS Foundation were ineligible to participate in the study. They were invited to a meeting to introduce the study. In total there were 28 youth facilitators and 37 club members who volunteered to participate in the focus groups (FGs).

### Data collection

The main data collection plan was to facilitate 12 FGs in all 7 local municipalities.

However, seven FGs were conducted until the team stopped as they were no longer finding new information. The FGs were conducted over 2 months (October and November 2014) in each of the seven local municipalities. The duration of each discussion was between 1 and 2 h. Both youth facilitators and volunteers participated in the same FG. Participants were interviewed in their respective local municipalities. A guide was used for the FGs to explore the socio-ecological factors influencing youth risk behaviour. The topics covered included the following: why young people are engaging in risky behaviours such as unsafe sex and substance abuse whilst there are numerous prevention interventions. The discussions included condom use, high teenage pregnancy and drug abuse. FGs were mainly conducted to develop a context-based youth risk reduction intervention in the context of OSS; therefore, all the youth facilitators and club members that volunteered in all the seven local municipalities participated. FGs were conducted in isiZulu by the first author, whilst a research assistant was employed to assist with the audio recording and note-taking.

### Data analysis

Framework analysis was used to analyse the data. The team considered it to be a better choice because it emphasises how both a priori issues and emergent-driven themes should guide the development of the analytic framework. This was something that fitted the aim of the study in that the team had certain predefined areas to explore but also wanted to remain open to discover the unexpected. Using the five stages of the framework analyses, all the team members initially listened to FGs, read transcripts and discussed emerging issues in the data, and a set of codes were identified. To ensure inter-rater consistency, the team compared and discussed their coding of transcripts. The data set was very large and focused much on youth experience. The team grouped numerous codes to form the categories. Bronfenbrenner’s social-ecological approach in health promotion was identified as a framework for the purposes of sifting and sorting rather than thinking about the data set in a more interpretative way. The transcripts were then organised into the framework category systematically applying the framework to each FG transcript using Nvivo v10 software. Indexed data for each category were summarised into a chart form; at this stage, the aim was to reduce the data set into a more manageable form. Having summarised all seven FGs in this way, the team was ready to move to the final stage of the framework analysis which involves finding patterns, pulling together key characteristics of the data to map and interpret the data set as a whole including description and clarification of concepts and representing the range and the nature of phenomena within data. In this study, validity was ensured through consistency of findings and through adequate and systematic use of the original data, for example using quotations from various FGs.

### Ethical considerations

The statement has been added after data analysis. Ethical approval was obtained from the University of KwaZulu-Natal Biomedical Research Ethics Committee (BREC) (REF: BE342/14). Permission was obtained from the Office of the Premier of KwaZulu-Natal. All who participated in the study provided written informed consent voluntarily. Most of the participants were reimbursed for their transport costs and we provided a finger lunch for all focus group participants.

## Results

### Study participants

The number of participants in each FG ranged from 6 to 12, with a total of 65. There were 15 men and 50 women aged 19–32 years, of whom 28 were facilitators (7 men and 21 women). The youth club volunteers comprised 37 young people who were working closely with the youth facilitators (Table 1).

**TABLE 1:** Socio-demographic characteristics of the participants.

Characteristics	Frequency
**Age (in years)**	
19–21	23
22–25	35
26 > 30	7
**Gender**	
Male	15
Female	50
**Number of facilitators**	28
**Number of volunteers**	37
**Number per local municipality**	
A	12
B	6
C	7
D	11
E	10
F	8
G	11

The study revealed the persistent individual and socio-ecological systems that play a role in the sexual risk behaviours of youth in uMgungundlovu District Municipality. The findings are presented as four broad themes (Table 2).

**TABLE 2:** Classification of main themes, sub-themes and codes.

Main themes	Sub-themes	Codes
**Individual factors**	Role modelling	Imitating older people Experimenting observed behaviour Learning from observation
	Gender stereotypes	Females lack backbone Males are hunters Mothered a child
	Decision-making	Decide to get pregnant Decide to date older men Decide not to go to the clinic No family planning use
**Microsystems**	Family	The role of the family Absent fathers The role of fathers Father as a disciplinarian Father’s dignity The role of mothers The role of siblings
	Friends	Peer pressure The role of friends
	Access to health services	Attitude of the nurses Girls do not go to clinic Nurses only help older females The role of the nurses
	Neighbourhood	Shebeens Schools Communities
	Relationships	Romantic relationships Relationship dynamics
**Exo-systems**	Economic status	Unemployment Poverty
	Transactional sex	Material things Sugar daddies Buy expensive clothes, phones
**Macrosystems**	Social norms	Culture Traditions Customs
	Policies	Alcohol regulations School polices Access to drugs and alcohol Consumption of alcohol

### Individual factors

The participants described individual factors such as role modelling and gender stereotypes that they felt led to the persistent exposure of youth to sexual risk behaviours. These are reported subsequently.

#### Role modelling

The majority of the participants agreed that younger learners, especially females from the primary schools, observe the behaviour of the high school learners, and during their adolescent stage, they model these behaviours. Both female and male participants echoed this explanation. One of the participants from FG 2 explained:

‘These young kids from the primary school see the older ones who are in high school, and think that oh, it means that when I reach that age, I would be having a right to date who ever I want to date, be with boys anytime, I will kiss, hug and have sex with them.’ (Female, youth facilitator, 22 years, FG 2)

In contrast to this, participants from FGs 4 and 5 talked about protecting factors among youth such as self-awareness as a motivation to abstain from high-risk behaviours:

‘Youth who know what they want and why they are going to school, those who know the risks of dating, their backgrounds and those with dreams, they don’t imitate other people’s behaviours because they do not want anything that will jeopardise their future as they are pursuing their short- and long-term goals.’ (Male, youth facilitator, 27 years, FG 5)

### Gender stereotypes and decision-making

The participants explained that youth risk-taking behaviour in their communities was influenced by poor decision-making and gender stereotypes. One participant expressed his concern regarding girls’ inability to decline unwanted sex:

‘Girls lack backbone, this is what is needed in them. They must learn how to say ‘no’ – not the ‘no’ that opens a way for a boy or a man to convince her. How can their partners control their future?’ (Male, youth facilitator, 21 years, FG 3)

In contrast, the actions of the boys were seen to be acceptable social norms. Some participants mentioned the natural and acceptable actions of males and they concluded that boys were said to be controlled by hormones and this becomes the basis for their engaging in sex without thinking:

‘It is a natural thing for boys to be hunters and there is nothing wrong … in searching for a girlfriend. … When sexually aroused, our brains stop working; our penises only direct us. I would sleep with whomever I meet. I am hunting because my penis is erect, not because I am in love with her.’ (Male, club member, 19 years, FG 5)

### Microsystem

The majority of the participants inferred that many sexual risk behaviours are related to the microsystem within which the individual functions. This includes their family (parents, siblings and extended family members), pressure from friends, lack of access to sex education and contraceptives in schools, the negative attitude of health workers, use of substances, and in particular alcohol, and their relationship dynamics with partners. These are further described subsequently.

### The role of parents

The dominant parental behaviour was strict discipline over their female children, which was reported to encourage risky sex. Some participants from FG 1 explained that parents’ strict discipline, lack of honest conversations about dating and sex, and limiting access to contraceptives for the girls were negative factors.

‘A parent will say, “I don’t want you to have a boyfriend”. So whatever a child is doing, she will keep it away from a parent, doing it on the side because she wants to do it and the parent will not know and she will hide it by all means. So going to a boy’s room, it is another practice of hiding from the parents, not knowing the risk.’ (Female, club member, 20 years, FG 1)

However, many participants from FGs 1, 2 and 7 felt that the involvement of the parents in their children’ lives serves as a protective factor.

‘These parents spend time with their children educating them about life skills, some of their children listen and do just what their parents are saying.’ (Female, youth facilitator, 20 years, FG7)

Single parents especially mothers were reported to not be in a position to raise a boy child whilst the presence of a father figure for boys was reported to promote positive behaviour in a boy child. A participant explained:

‘The only person that children finding it easy to talk to about the issues in general are their mothers, because the father has authority, [*and*] it is hard to just talk to him.’ (Female, club member, 26 years, FG 1)

Other participants felt that women in their society are not socialised to raise boys and one of the participants explained:

‘The other problem is that there are children that are raised by a single parent, especially those boys that are raised by their mothers. It is not easy for boys to speak to their mothers about manhood. On the other hand, fathers were reported to be helpful in nurturing and guiding a male child.’ (Male, club member, 31 years, FG 2)

#### The role of the siblings and extended family members

Some siblings and extended family members were reported to be influencing sexual risk behaviours targeted in this study. Participants mentioned sisters as having a positive influence on their younger sisters, whilst some uncles were perceived to be facilitating exposure to risky behaviours. A participant had this explanation:

‘…Because she knows that, once her sister is aware of her actions or behaviour, she will tell their mother and she will be in trouble. Another participant was concerned about the negative role uncles play in facilitation of exposure to risky behaviours.’ (Female, club volunteer, 22 years, FG 6)

‘The uncles send kids to buy alcohol and even if a child is buying it for himself, nobody will notice. Another problem with uncles is that they have multiple sexual partners and sometimes these relationships result in pregnancy and violence.’ (Female, youth facilitator, FG 2)

#### Friends: The role of peer pressure

Different perspectives on peer pressure among the youth were reported. After lengthy discussions regarding the extent of peer pressure in increasing the high-risk behaviours among young people, the majority of the participants agreed that resistance to peer pressure depends upon the individual. However, participants further reported that peer pressure was prevalent within the same gender. The following comments explain their views on the current levels of peer pressure among young people. A participant from FG 5 explained:

‘Young girls from age 15 or 16 years have children, so if you are the only one without a kid, they will say, “You don’t have a child” Are you not a mom? “We all have kids”. Friends will pressurise you to get pregnant.’ (Female, youth facilitator, 24 years, FG 5)

In contrast, participants also reported that boys were having sexual intercourse only to conform. One of the participants from FG3 gave this example:

‘Not that boys are always longing for sex. Friends sometimes influence boys. For instance, X may tell me that “I have had sex with my girlfriend” but in my case, I have not yet been doing it with my girlfriend. I will then be under pressure to do it.’ (Male, club member, 23 years, FG 3)

#### Attitude of health care workers: Access to health services

The youth facilitators confirmed health workers’ negative attitudes to girls’ need for contraceptives, as well as the limited access to and knowledge about contraceptives, as reasons young people in uMgungundlovu find themselves engaging in unsafe sex, particularly observed among young girls who are still at high school.

It emerged from FGs 1, 2, 4 and 6 that clinic nurses prevent girls who are sexually active from initiating or accessing family planning services; because of their fear of being shamed and reported by nurses for engaging in sex, they end up not going to the clinic. A participant from FG 1, who came from a community with only one clinic, provided the following narrative:

‘I think the clinic has something to do with this because they serve adults only. So the girls found it difficult to go to the clinic because they would not get any help. Instead, they get some questions like, “What are you doing?” and “Why are you preventing at your age?” So that is why they decide not to go to the clinic at all.’ (Female, youth facilitator, 32 years, FG 1)

Another participant from FG 4 gave the following explanation:

‘In some cases, girls are scared to go to the clinic because maybe a nurse who is working there is staying near her home, so she assumes that the nurse will report to her parents that she saw her at the clinic doing family planning.’ (Female, youth facilitator, 27 years, FG 4)

Besides their critical attitudes, the issue of nurses’ personal moral stance was reported to create an environment in which they share only limited sex education information with boys. The participants reported this as an issue, which contributes to poor decision-making when it comes to safe sex. A male participant from FG 6 had this to say:

‘When I go to the clinic, I want help with information because I made a mistake I got someone pregnant. The nurse does not give me information because she is afraid because she says, “I go to church with his grandma. People will say I am talking about penises with children” and she decides not to give me information, which is not right because young people need information.’ (Male, club member, 20 years, FG 6)

#### The role of the neighbourhood in alcohol use

Most of the time when participants were asked about the context that leads to young people participating in unsafe sex, being intoxicated was one of the responses mentioned by participants. This was prevalent across all the groups. Being drunk was reported to put girls at risk of being raped and having unprotected sex. FG 4 participants gave these examples:

‘Some clubs reserve rooms where clients are allowed to do sex, they go there taking turns. When they arrive in these clubs, girls drink until they get drunk and then they take them to that room. There are so many things happening in the clubs, so in the morning, you wake up not knowing what happened.’ (Female, club member, 20 years, FG 4)

Further, in this study, girls were reported to be loose and boys’ behaviour was treated as something normal and acceptable. Another participant from FG 4 explained:

‘Let us say maybe someone knows that there is a party and there is free alcohol, she must now do sex with the boys she just met. Because she is drunk, she does it without being aware.’ (Male, club member, 23 years, FG 4)

#### The role of sexual partner, relationships and their dynamics

In this study, most of the participants felt that young girls are constantly involved in romantic relationships that influence their behaviours negatively. The need to gain or prove a partner’s trust and sexual satisfaction emerged as some of the main factors that influence the girls to participate in unsafe sex. Some participants felt that girls were encouraged by their partners to discontinue using the injectable contraceptives, as these led to unpleasant sexual experiences. Because of the need to satisfy their partner and to gain their trust, unsafe sex would then result or intimate partner violence. This pattern of behaviour was noted in most of the FGs. Below are comments from FG 1 and FG 4 regarding unsafe sex:

‘Another thing when you are using the injection to prevent pregnancy, he will say he doesn’t like a girl who uses injections because during sexual intercourse, she gets wet [*the vagina becomes full of the fluids*].’ (Female, youth facilitator, 21 years, FG 1)

‘He tells you that he is dating you only.’ (Female, club member, 25 years, FG 4)

In contrast, some participants reported physical abuse that was experienced by the females that was perpetrated by their partners when they insist on condom use. A female participant said:

‘If you refuse to do sex without a condom, they force themselves physically. The participants noted that the community leadership provided poor examples when they engage in sexual relationships with young people and had many concurrent partners.’ (Female, club member, 23 years, FG 2)

### Exo-system

#### Economic status of the community

Some participants reported that the youth in the area where they facilitate youth programmes live in poverty because of the high unemployment rate. This was mentioned as a reason why girls become involved in transactional sex relationships.

‘The problem we’re having here is that unemployment rates are high; another thing is that children end up dating these old men because they want food, because of poverty.’ (Male, youth facilitator, 31 years)

The need to have material things such as cell phones and clothes similar to one’s friends was seen to lead to girls dating men for money, which in most cases required that they reciprocate by having sex. This was truer for young girls than boys. For example, a participant from FG 4 stated this:

‘Maybe when I see that my friend is in a relationship with someone who gives her money. He has the right car, he always drops my friend with plastics with clothes, she has an expensive phone, and she wears nice clothes, all those things. I see myself that no, I’m not in style if I don’t get the right person who is working. I then end up getting someone who is working because I want to be like that person who is the same age as me. Maybe that person is old and at the end I get hurt.’ (Female, youth facilitator, 23 years, FG 4)

### Macrosystem

#### Social norms and government policies

**Social norms:** In this study, participants reported that the current social norms influence health outcomes negatively. These emphasise the acceptable practice of multiple sexual partners. Some participants mentioned social norms as the core as to how black Africans approach sexual relationships. Participants from FG 5 stated this:

‘Our social norms have destroyed us as black people. We are playboys. If you have one girlfriend, they see you as a bachelor.’ (Male, youth facilitator, 33 years, FG 5).

‘Our leaders should be the role models. How many wives and girlfriends do they have? They marry women of similar age to their granddaughters. We have a problem of the social norms and socialisation, which produces bad consequences.’ (Male, club member, 34 years, FG 5)

In addition to such social norms, one of the participants revealed that the value attached to marriage in Zulu culture encourages girls to participate in unprotected sex where they could fall pregnant, as that supposedly guarantees marriage:

‘Another thing, young women fall pregnant and to them that shows that you are a decent person if you are married. They believe you are nothing if you are not married; that is why they are sleeping with men because they want this marriage.’ (Female, youth facilitator, 22 years)

However, the majority of the participants countered this belief as they felt that it was lacking substance. A participant from FG 4 explained:

‘Sometimes you find out that the boy is also attending school, he doesn’t have money to pay lobola [*bride wealth*], or the partner is working but he doesn’t marry her. There are many children that are born out of wedlock in our days.’ (Female, youth facilitator, 26 years, FG 4)

**Laws related to alcohol use:** As presented above, alcohol use featured as one of the reasons that young people participate in unsafe sex. According to the FG participants, alcohol is easily accessible for the youth of uMgungundlovu Municipality. The non-adherence to the laws was mentioned as encouraging youth in this area to indulge in consumption of alcohol. One of the participants from FG 6 related this encounter:

‘In this township, there are ten taverns and only three have legal documents.’ (Male, youth club member, 32 years, FG 6)

This picture emphasises the poor implementation of the alcohol regulations, which indicates the level of alcohol availability and the extent of the consumption of alcohol.

## Discussion

Historically in South Africa, most of the face-to-face interventions for sexuality education have been concentrated in high schools and mostly towards those aged 14 years and older who are regarded as the vulnerable group.^21^ However, our results show that these efforts might be too late, as young people are exposed to high-risk behaviours early through exposure to community activities and social norms.^31^ The findings of our research confirm the harmful social norms and the acceptance of the male’s dominant role in our society that perpetuates gender inequality to the detriment of females.^32^ Although the South African Constitution entrenches gender equality,^33^ these negative harmful norms are upheld by society and place young girls in a subservient role and at risk of unplanned pregnancy,^4^ HIV and other sexually transmitted infections.^4^ Males are socialised in an environment that promotes early sexual initiation and multiple sexual partners, whilst females are socialised to be submissive.^34^

Whilst some interventions have been reported as having positive health outcomes,^1^ usually change in behaviour is not sustainable, owing to the environmental risk factors including community norms.^35^ Young people function within a microsystem, which is defined by their families, friends and neighbourhood.^30^ Targeting the social systems to which young people are exposed day and night might contribute to the efforts aiming at reducing sexual risk behaviours. An increase in support by parents, siblings and peers may lead to stronger predictors of positive health. For more than a decade, the negative attitudes of the nurses towards young people who seek family planning and HIV testing services have been in the spotlight. ^36,37,38^ Although, in South Africa, policies to improve access to reproductive health services to youth are in place, reports of the negative attitudes of the nurses continue to surface.^39^ In the study, it was reported that youth were reluctant to attend family planning clinics owing to the attitude of the nurses although they were dating and knew the importance of contraceptives. Access to family planning in uMgungundlovu municipality continues to be problematic for young people.

We also found high levels of alcohol abuse in this study. Our results are congruent with existing literature on youth risk behaviours, gender-based violence^7,34^ and violence.^40^ For instance, previous studies show that people who abuse alcohol are at risk of becoming victims or perpetrators of rape.^41^ Whilst there are existing interventions aiming at curbing drug abuse, the prevalence of alcohol abuse continues to be high among young people,^42,43^ and the association between alcohol abuse and intimate partner violence is also prevalent among young people.^44^ The rules and regulations of alcohol use are in existence in South Africa. However, implementation and monitoring of these policies is lacking.^42,43^

This study confirms the existence of gender imbalances in the romantic relationships of the young people. These include intimate partner violence, infidelity and lack of condom use, which are similar to those of the older people around them.^45^ Targeting young people only is not producing fruit;^15,46^ rather new interventions must aim to address the community norms influencing youth risk behaviours. The other important factor influencing sexual risk behaviour among the youth that has been identified in the literature is unemployment. In South Africa, the official unemployment rate was 27.1% in 2016.^47^ Since 2014, approximately 60.5% of South African youth aged between 19 and 24 years were residing in low-income households. Although this phenomenon was documented previously, there has been no change in the status quo and our results are congruent with the existing evidence.^48^ Owing to poverty levels, some South African youth aim to ‘love’ strategically, in order to negotiate themselves out of poverty and marginalisation,^49^ because current government policies are not accommodating those young people who are unemployed.

Presently, the government is providing child support grants for young mothers, an amount of R380 monthly in 2017.^3^ In some families, this is the only source of income^3^. Livelihood activities to support families are required for the mitigation of poverty. Despite participants in our study reporting how the girls use pregnancy in the hope of getting married, marital rates began to drop in the 1960s, because young men were unable to pay *ilobola* (bride wealth) and establish an independent homestead for their family.^50^ The high unemployment rate in South Africa where a quarter of those seeking work are unable to find work further disadvantages payment of *ilobola* and marriages.^50^ Cultural customs in African society are prevalent and they describe the characteristics of the community. Our study shows that some customs are influencing the behaviour of the youth negatively. For instance, community leadership has been blamed for having multiple partners and sexual relationships with young people. This behaviour contradicts efforts that have been implemented towards reducing sexual risk behaviour among youth. However, reduction in the number of partners owing to the high HIV prevalence in KwaZulu-Natal is vital.

## Strength and limitations

The study only included young people to ensure their voices are heard. Young people shared their feelings and experiences and how they see current interventions and situations that they experienced in their context. This study was localised to a district municipality.

## Conclusions

This article contributes to the literature on the sexuality education of the youth in South Africa by demonstrating various socio-ecological factors influencing youth sex risk behaviours in uMgungundlovu district. Interventions addressing the risk behaviour of the young people in uMgungundlovu Municipality need to consider all these systems, because interventions targeting individuals have to date not resulted in the desired health outcomes. This article proposes an ecological theory, which focuses attention on both the individual and the social environment. The theory assumes that appropriate changes in the social environment will produce changes in individuals and that support of individuals in the population is essential for implementing environmental changes. This would require a multi-sectoral response by the provincial government to address the many issues raised in these FGs.
